# Multiple-Tissue Integrative Transcriptome-Wide Association Studies Discovered New Genes Associated With Amyotrophic Lateral Sclerosis

**DOI:** 10.3389/fgene.2020.587243

**Published:** 2020-11-20

**Authors:** Lishun Xiao, Zhongshang Yuan, Siyi Jin, Ting Wang, Shuiping Huang, Ping Zeng

**Affiliations:** ^1^Department of Epidemiology and Biostatistics, Xuzhou Medical University, Xuzhou, China; ^2^Department of Biostatistics, School of Public Health, Cheeloo College of Medicine, Shandong University, Jinan, China; ^3^Center for Medical Statistics and Data Analysis, School of Public Health, Xuzhou Medical University, Xuzhou, China

**Keywords:** transcriptome-wide association study (TWAS), amyotrophic lateral sclerosis (ALS), genome-wide association studies (GWAS), brain tissue, type I error control

## Abstract

Genome-wide association studies (GWAS) have identified multiple causal genes associated with amyotrophic lateral sclerosis (ALS); however, the genetic architecture of ALS remains completely unknown and a large number of causal genes have yet been discovered. To full such gap in part, we implemented an integrative analysis of transcriptome-wide association study (TWAS) for ALS to prioritize causal genes with summary statistics from 80,610 European individuals and employed 13 GTEx brain tissues as reference transcriptome panels. The summary-level TWAS analysis with single brain tissue was first undertaken and then a flexible *p*-value combination strategy, called summary data-based Cauchy Aggregation TWAS (SCAT), was proposed to pool association signals from single-tissue TWAS analysis while protecting against highly positive correlation among tests. Extensive simulations demonstrated SCAT can produce well-calibrated *p*-value for the control of type I error and was often much more powerful to identify association signals across various scenarios compared with single-tissue TWAS analysis. Using SCAT, we replicated three ALS-associated genes (i.e., *ATXN3*, *SCFD1*, and *C9orf72*) identified in previous GWASs and discovered additional five genes (i.e., *SLC9A8*, *FAM66D*, *TRIP11*, *JUP*, and *RP11-529H20.6*) which were not reported before. Furthermore, we discovered the five associations were largely driven by genes themselves and thus might be new genes which were likely related to the risk of ALS. However, further investigations are warranted to verify these results and untangle the pathophysiological function of the genes in developing ALS.

## Background

Amyotrophic lateral sclerosis (ALS), also known as Lou Gehrig’s disease, is an adult-onset progressive and fatal neurodegenerative disease (Kiernan et al., 2011). Although its prevalence rate is not high worldwide (Vazquez, 2008; Marin et al., 2017; Mehta et al., 2018), ALS can lead to severe clinical consequence (Chio et al., 2009) and economic burden (Larkindale et al., 2014; Gladman and Zinman, 2015). One of the greatest challenges with regards to ALS is that few effective therapeutic interventions have been confirmed and nearly no cure is available in clinic (Mehta et al., 2018; Zeng et al., 2019a). In addition, it is evaluated that the ALS cases across the globe will elevate up to ∼400K in the coming 20 years owing to aging of the population (Arthur et al., 2016), which will further aggravate the socioeconomic threat of ALS.

Prior work has revealed that ALS is highly heritable, with the heritability ranging from 0.52 (95%CI 0.43–0.62) for the ordinary population, to 0.37 (95%CI 0.20–0.54) for those without genetic risks according to population-based studies, and to 0.66 (95%CI 0.59–0.74) based on mother-daughter pairings (Ryan et al., 2019) or 0.61 (95%CI 0.38–0.78) in terms of twin studies (Al-Chalabi et al., 2010). Therefore, understanding the genetic etiology of ALS and identifying risk genes are crucial for early prevention and also have the potential to discover effective therapeutic targets. Indeed, in the past decade dozens of genome-wide association studies (GWAS) have identified multiple single nucleotide polymorphisms (SNPs) and genes causally associated with ALS (McMahon et al., 2019) (Table 1 and Supplementary Table S1). However, the genetic architecture of ALS remains largely unknown and the functional influences of those genetic variants are also not completely clear. For example, the SNP-based heritability estimated in GWAS is only 21%, which is much smaller than that reported above (Keller et al., 2014), implying a large amount of causal genes have not yet been identified and the effort to find causative genes for ALS should continue.

**TABLE 1:** Previous association studies for ALS in terms of the GWAS catalog.

Year	Pop	cases/controls (discover + replication)	*m*	References
2007	EUR	276/271	3	Schymick et al., 2007
2007	EUR	461/450 + 876/906	1	Van Es et al., 2007
2007	EUR	221/211 + 737/721	1	Cronin et al., 2007
2008	EUR	737/721 + 1,030/1,195	3	Van Es et al., 2008
2009	EUR	958/932 + 309/404	1	Cronin et al., 2009
2009	EUR	1,821/2,258 + 538/556	14	Landers et al., 2009
2009	EUR	2,323/9,013 + 2,532/5,940	3	van Es et al., 2009
2010	EUR	405/497	4	Laaksovirta et al., 2010
2010	EUR	4,857/8,987	0	Shatunov et al., 2010
2010	EUR	639/6,257 + 183/961	2	Kwee et al., 2012
2013	EUR	4,243/5,112	19	The Alsgen Consortium, 2013
2013	EUR	6,100/7,125 + 2,074/2,556	3	Fogh et al., 2013
2014	EUR	4,377 + 435/14,431 + 4,056/3,958	10	Diekstra et al., 2014
2015	EUR	25/1,179	1	McLaughlin et al., 2015
2016	EUR	12,577/23,475 + 2,579/2,767	4	van Rheenen et al., 2016
2018	EUR	20,806/59,804 + 4,159/18,650	10	Nicolas et al., 2018
2019	EUR	4,244/3,106	1	Dekker et al., 2019
2013	CHI	506/1,859 + 706/1,777	4	Deng et al., 2013
2013	CHI	4,243 (age of ALS on-set)	15	The Alsgen Consortium, 2013
2013	CHI	250/250	174	Xie et al., 2014
2016	CHI	94/376	1	Chen C.J. et al., 2016
2017	CHI	1,234/2,850 + 576/683	7	Benyamin et al., 2017

*Pop denotes which populations the GWAS was performed on, with EUR representing the European population and CHI representing the Chinese Han population; the third column is the sample size of GWAS in the discover stage and in the replication stage if conducted; *m* denotes the number of unique genes mapped by associated SNPs; these results are overviewed in terms of the GWAS catalog at https://www.ebi.ac.uk/gwas (until 2020-02-02). Of note, some of GWASs had only limited sample sizes, which might influence the validity of the discovered genetic variants and mapped genes in these studies. Therefore, the associations need to interpret in caution.*

**FIGURE 1:**
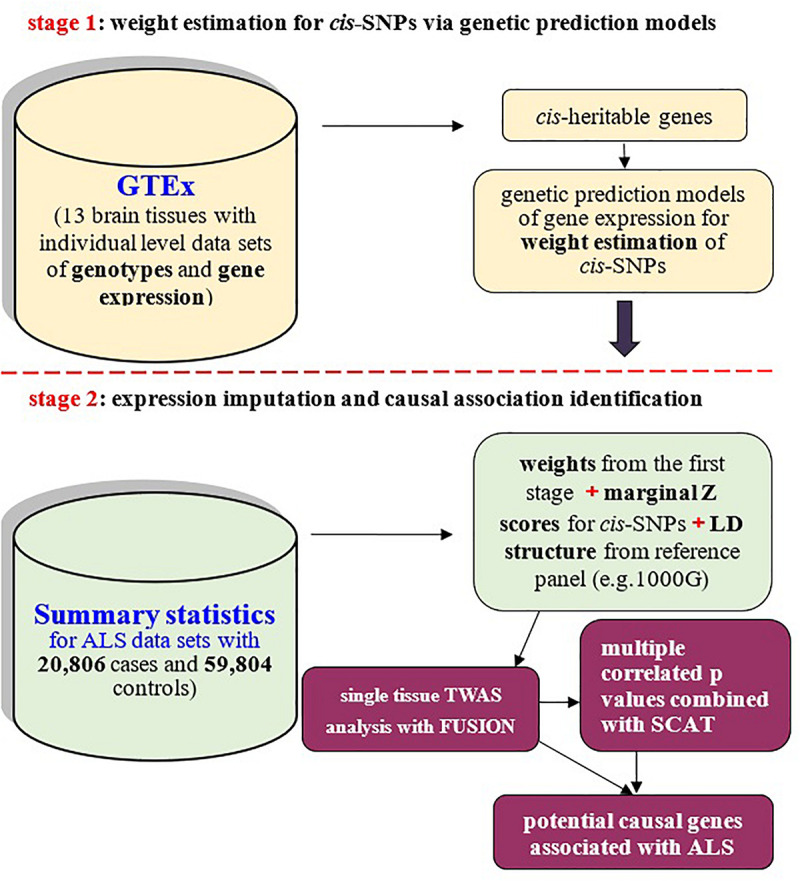
Schematic framework of TWAS with FUSION and SCAT based on only summary-level datasets and reference panel for linkage disequilibrium (LD) structure of SNPs. TWAS can be viewed to be a relatively independent two-stage inference procedure: the first stage is to estimate weights for *cis*-SNPs with GTEx brain transcriptome reference panel (the **top panel**); the second stage is to examine causal association between genes and ALS with weights obtained from the first stage (the **bottom panel**).

The importance of gene expression regulation in complex diseases motivates us to apply novel statistical tools prioritizing causal genes of ALS through the integration of expression quantitative trait loci (eQTL) into GWAS (Nica et al., 2010; Nicolae et al., 2010; GTEx Consortium, 2015; Li et al., 2016; Wen et al., 2016; GTEx Consortium, 2017; Mancuso et al., 2019). Transcriptome-wide association study (TWAS) is exactly one of such approaches popular in genomic integrative analysis (Gusev et al., 2016; Hu et al., 2019; Mancuso et al., 2019; Wainberg et al., 2019). Methodologically, TWAS can be viewed as a relatively independent two-stage inference procedure to discover causal genes (Figure 1). Briefly, in the first stage weights (i.e., the joint effect sizes) of *cis*-SNPs of a given gene are computed from external tissue-related transcriptome reference datasets; and then the association between the imputed expression and the disease of interest is examined for that gene in the second stage. The original TWAS analysis needs large scale individual-level data sets (Gusev et al., 2016), which limits its applicability due to unavailability of such data sets because of privacy concerns in data sharing among various research groups (Gusev et al., 2016; Pasaniuc and Price, 2016). Fortunately, such limitation is already eliminated with the development of summary-level TWAS (Gusev et al., 2016; Barbeira et al., 2018), for which only pre-estimated weights of QTL and summary statistics of GWAS are necessary.

Moreover, because it has been shown that spurious associations may be generated if integrating gene expression from tissues that are not biologically related to the disease (Wainberg et al., 2019), a strongly recommended strategy in TWAS analysis is that one should calculate weights of *cis*-SNPs with expression measurements from the most relevant tissues in the first stage. For instance, the breast-cancer TWAS analysis employs transcriptome datasets of the breast tissue (Wu et al., 2018) and the prostate-cancer TWAS analysis applies transcriptome datasets of the prostate tissue (Mancuso et al., 2018; Wu et al., 2019). Therefore, it is the natural choice of brain tissues when implementing TWAS for ALS. There are 13 GTEx brain tissues that can be employed as reference transcriptome panels (GTEx Consortium, 2015, 2017) (Table 2). The rich transcriptome datasets offer an unprecedented opportunity to comprehensively integrating QTL information into the GWAS of ALS. In the meantime, they also propose a great statistical challenge for such integration.

**TABLE 2:** ALS-associated genes identified by SCAT or FUSION with 13 GTEx brain tissues.

Tissue	*N*	*p*_0_	*p*_1_ (%)	*FAM66D*	*C9orf72*	*TRIP11*	*RP11-529H20.6*	*ATXN3*	*JUP*	*SCFD1*	*SLC9A8*
Amygdala	81	1,799	0 (0.00)								2.85E-1
Anterior cingulate cortex BA24	102	2,653	4 (0.15)				1.20E-1	4.11E-3		6.90E-4	
Caudate basal ganglia	126	3,586	1 (0.03)		2.71E-8			3.06E-1			
Cerebellar hemisphere	113	4,327	6 (0.14)	3.36E-1	3.93E-10	2.01E-1	2.37E-1	3.45E-1		7.25E-4	4.76E-2
Cerebellum	137	5,752	4 (0.07)		4.97E-4	5.86E-3		1.02E-2		1.15E-3	3.73E-1
Cortex	119	3,943	3 (0.08)	7.79E-3	6.41E-3	2.00E-1	1.22E-1				2.00E-1
Frontal cortex BA9	104	3,080	1 (0.03)	5.87E-1	3.84E-16					1.88E-1	
Hippocampus	99	2,245	1 (0.04)	3.66E-1	1.12E-4			8.61E-2			8.61E-2
Hypothalamus	98	2,257	3 (0.13)	4.94E-1			3.65E-1	3.65E-1	1.82E-2	1.55E-4	6.40E-3
Nucleus accumbens basal ganglia	114	3,172	2 (0.06)	5.53E-1	3.32E-24		4.91E-3				
Putamen basal ganglia	98	2,766	1 (0.04)		6.04E-7		2.07E-1				
Spinal cord cervical c-1	76	1,974	2 (0.10)	4.97E-1	1.26E-7						
Substantia nigra	70	1,568	2 (0.13)								
SCAT		11469	8 (0.07)	4.22E-2	1.08E-22	3.49E-2	4.10E-2	3.68E-2	4.22E-2	1.20E-3	4.22E-2

**N* is the sample size of gene expression in each tissue; *p*_0_ denotes the number of converged genes with heritability estimation, *p*_1_ (%) is the number (or proportion) of associated genes that have FDR < 0.05 in each tissue before adjustment of the 13 GTEx brain tissues.*

Performing ALS TWAS analysis from one brain tissue to another and then adjusting for multiple comparisons is a conventional approach. However, doing this may be underpowered because of the multiple testing burden; and such a manipulation is not optimal as it ignores useful information of shared eQTLs across brain tissues (GTEx Consortium, 2017). Therefore, it is important to integrate associations from all available brain tissues in the TWAS analysis of ALS with a more efficient manner, which would have the potential to improve power and discover newly genes associated with ALS. However, in terms of our literature view there is little existing work on how to aggregate such evidence efficiently when only summary-level eQTL and GWAS marginal statistics are utilizable. It is hence desirable to construct feasible omnibus tests to handle this problem.

The Fisher’s method (Fisher, 1934), one commonly used omnibus test, may be the first choice. Unfortunately, the Fisher’s method is only valid for independent multiple tests and thus cannot be employed due to highly positive correlation among individual TWAS tests (see simulations below for details). In fact, as we will demonstrate later, the Fisher’s method is overinflated and can lead to too many spurious associations when the TWAS test statistics are not independent. Alternatively, one may take the minimum *p*-value as the significance measure (Conneely and Boehnke, 2007). However, due to the same issue of unknown positive dependence, the null distribution of the minimum *p*-value may be extremely complicated and the computation is often time-consuming since numerical permutation/bootstrap is involved (Conneely and Boehnke, 2007; Sun and Lin, 2019).

Therefore, it is of substantial interest to develop omnibus tests that are robust against correlation. To achieve this objective, herein we propose a novel *p*-values integrative strategy called summary data-based Cauchy Aggregation TWAS (SCAT). Compared to previous approaches, SCAT owns an attractive strength that it takes the summary of a set of *p*-values as test statistic and evaluates the significance analytically without the knowledge of correlation structure. Consequently, SCAT is extraordinarily flexible and computationally fast. With extensive simulation studies we demonstrated that SCAT can produce well-calibrated *p*-value for the control of type I error and is often much more powerful compared with single-tissue TWAS analysis. Finally, using SCAT we discovered several new ALS-associated genes that would be missed by existing statistical strategies.

## Materials and Methods

### GWAS Summary Statistics for ALS

We obtained marginal summary statistics (e.g., *Z* scores) of ALS from the largest ALS GWAS to date (Nicolas et al., 2018). This study included several previous ALS cohorts such as the work of van Rheenen et al. (2016). For each SNP the logistic regression was first implemented per cohort with individual-level genotypes while incorporating several top principal components, age, and gender as covariates. Then, the inverse-variance weighted fixed-effect meta-analysis was implemented to pool association results across cohorts. Finally, after quality control approximately 8.6 million SNPs on 20,806 cases and 59,804 controls of European ancestry were left for our TWAS analysis.

### TWAS Analysis With Single Brain Tissue

To be self-contained, we first introduce TWAS approach for individual-level dataset. Suppose that **G** is an *n* × *m* matrix of genotypes of *cis*-SNPs for a gene, *n* is the sample size for ALS and *m* is the number of genetic variants and generally changes from gene to gene; **E** is an *n*-vector for *unmeasured* gene expression in the ALS GWAS and **y** is an *n*-vector of binary variable for ALS cases and controls. In addition, assume **g** is a *d* × *m* genotype matrix of *cis*-SNPs and **e** is a *d*-vector of gene expression from one of the GTEx brain tissues for the same gene, with *d* the sample size of the reference panel. The individual-level TWAS analysis can be implemented as

(1) stage⁢ 1:_⁢weights⁢estimation⁢with⁢genetic⁢prediction⁢models_⁢e=fw⁢(gw)⇒w^stage⁢ 2:_⁢gene⁢expression⁢imputation⁢and⁢association⁢analysis_⁢logit⁢(μ)=E^⁢θ⁢with⁢E^=G⁢w^

where **w** = (*w*_1_, *w*_2_, …, *w*_*m*_) is the *m*-vector of effect sizes for *cis*-SNPs and can be estimated (denoted by $\hat{\text{w}}$) with some genetic prediction model (denoted by *f***_*w*_**) (Zeng and Zhou, 2017); **ε** is a normal residual and **μ** is the expectation of **y**; and θ is the effect size for imputed gene expression. In the TWAS analysis we aim to test for the null hypothesis *H*_0_: θ = 0. It is seen that TWAS bridges the gap between QTL and GWAS in a conceptually simple fashion.

### FUSION: A Summary-Level TWAS With Single Tissue

When only summary-level datasets are available (as the case in our analysis of ALS), under the condition of no association between SNP and ALS we have

(2) $$\left\{ \begin{array}{c} {\hat{\text{z}}}^{\text{ALS}}\,\_\,\text{∼}\,\text{MVN}\,\left(0,R\right)\\ {\hat{\text{z}}}^{\text{ALS}}\,{\hat{\text{w}}}^{T}\,\_\,\text{∼}\,\text{MVN}\,\left\{0,\hat{\text{w}}\,\text{R}\,{\hat{\text{w}}}^{T}\right\} \end{array}\right.$$

where ${\hat{\text{z}}}^{\text{ALS}}$ is an *m*-vector of marginal *Z* scores of *cis*-SNPs and often generated with single SNP regression (Zeng et al., 2015); **MVN** denotes the multivariate normal distribution, and **R** is the unknown LD correlation matrix among *cis*-SNPs and can be approximately estimated with reference datasets such as 1000 Genomes Project (The 1000 Genomes Project Consortium, 2015). With these in hand we define the TWAS statistic as

(3) $$Z_{t}=\{{\hat{\text{z}}}^{\text{ALS}}{\hat{\text{w}}}^{T}\text{\}}\{\hat{\text{w}}\text{R}{\hat{\text{w}}}^{T}{\text{\}}}^{-\frac{1}{2}}$$

The *p*-value of *Z*_*t*_ can be easily obtained since it asymptotically follows a standard normal distribution. The above TWAS analysis is implemented through the FUSION software (Gusev et al., 2016).

### Summary-Level TWAS for Multiple-Tissues With Known Correlation Structure

When the correlation structure among gene expressions is known (but it is in fact unknown), a summary-level TWAS approach combining FUSION results of multiple tissues can be designed assuming no association between the gene and ALS across tissues

(4) $$Q\,\_={\text{ZC}}^{-1}\,{\text{Z}}^{T}∼\chi_{T}^{2}$$

where **Z** = (*Z*_1_, …, *Z*_*T*_) approximately follows **MVN**(0, **C**) with **C** the correlation matrix of gene expressions from *T* tissues. The above method is also called multiXcan (Barbeira et al., 2019) and provides an omnibus test for the combination of effect in any brain tissue while accounting for correlation. We refer to the test shown in (4) as the *oracle* TWAS. However, due to the lack of transcriptome reference panels (The 1000 Genomes Project Consortium, 2015), **C** is often unknown or cannot be estimated accurately from expression datasets with small sample sizes (Gusev et al., 2016; GTEx Consortium, 2017).

### Combination of TWAS via the Aggregated Cauchy Association Test

We here introduce how SCAT can be adopted in our ALS TWAS analysis. First, we separately implement FUSION for each brain tissue and yield *Z*_*t*_ and *p*_*t*_ (*t* = 1, 2, …, *T*; with *T* = 13 here); as expected, these *p*_*t*_s (or *Z*_*t*_s) are highly correlated (see also below) (Brown, 1975; Kost and McDermott, 2002; Poole et al., 2016; Heard and Rubin-Delanchy, 2018). As a result, as mentioned before the Fisher’s method, which assumes independent tests, is not appropriate. We instead apply SCAT which allows us to aggregate multiple potentially dependent *p*-values obtained from multiple FUSION analyses into a single well-calibrated *p*-value that can maintain the type I error correctly. The pooled *p*-value of SCAT follows a Cauchy distribution regardless whether *p*-values are correlated or not (Liu et al., 2019; Liu and Xie, 2019). Briefly, with SCAT we have

$$T_{SCAT\_}=\sum_{t=1}^{T}\varpi_{t}\tan\left\{\left(\frac{1}{2}-p_{t}\right)\text{π}\right\}$$

(5) $$PT_{SCAT\_}=\frac{1}{2}-\text{arc tan}\left\{T_{SCAT}/\left(\sum_{t=1}^{T}\varpi_{t}\right)\right\}/\text{π}$$

where ϖ_*t*_ denotes the non-negative weight for each *p*_*t*_ with $\sum_{t=1}^{T}\varpi_{t}=1$, and assume that ϖ_*t*_ is independent of *p*_*t*_. When no prior information is available, equal weights are utilized. Because SCAT only takes a group of *p*-values as input and no any dependence structure is required, its implementation is thus rather straightforward and fast.

### Numerical Simulations

We implement simulation studies to assess the performance of SCAT and compare it with the Fisher’s method. As described before because both the two methods used only *p*-values as input; we thus start our simulations by generating a series of independent or non-independent *p*-values. This is also the simulation framework used in previous work (Liu and Xie, 2019). Specifically, we first obtained the correlation matrix of **Z** values of FUSION (i.e., the **C** matrix; shown in Supplementary Figure S1) and generated a 13-dimentional multivariate random variable which followed **MVN**(**μ**, **C**). Then, we yielded the *p*-value for each marginal random variable by assuming it followed a standard normal distribution. Finally, we combined these *p*-values with SCAT or the Fisher’s method.

We set μ = 0 when evaluating the type I error control, but randomly sampled **μ** from an independent normal distribution with mean zero and variance 2.5 when assessing the statistical power. A total of 10^6^ or 10^3^ replications were generated for type I error control and power evaluation respectively. Furthermore, to match the application in real-life datasets — not all genes were identified to be *cis*-heritable across all brain tissues with the current sample sizes of transcriptome datasets (see Supplementary Figure S2 for more information) — in each replication of the power assessment we randomly selected at least five but at most eleven tissues to be missing. Doing this was equivalent to generating missing values in each group of marginal *p*-values.

In the present analysis genes with false discover rate (FDR) (Benjamini and Hochberg, 1995) less than 0.05 were defined to be associated genes. All analyses were carried out with the R software (version 3.6.2); and the codes to reproduce simulations as well as the FUSION results of ALS can be found at https://github.com/biostatpzeng. In addition, since we only employed summary-level genetic datasets that can be publicly available; therefore, additional ethical review was not needed for our study.

## Results

### Type I Error Control and Power Evaluation

**FIGURE 2:**
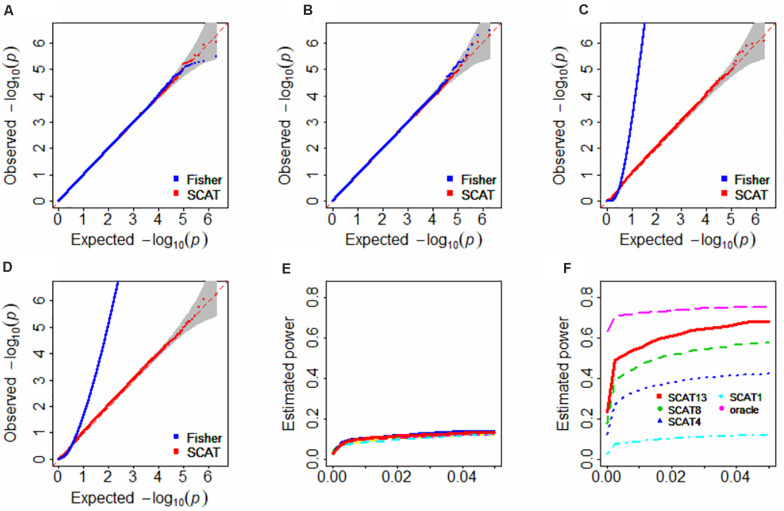
Type I error control **(A–D)** and Estimated statistical power **(E,F)** in the simulation studies. In **(A,B)**, the correlation matrix was independent; in panels **(C,D)**, the correlation matrix was specified with the matrix shown in Supplementary Figure S2; in **(E)**, the clustered lines with various colors represent the 13 types of FUSION analysis with one tissue and cannot be clearly separated; in **(F)**, the number attached by SCAT indicates various tissues included; oracle denotes the oracle TWAS approach with the matrix shown in Supplementary Figure S2; because the inclusion of all 13 tissues in the oracle TWAS would result in 100% power; thus, here we only considers three tissues that were randomly selected in the oracle TWAS.

It is observed that both the Fisher’s method and SCAT can correctly control the type I error if the *p*-values are independent (Figures 2A,B). However, in the presence of positive dependence among *p*-values, the Fisher’s method fails to maintain the type I error control and is rather liberal (Figures 2C,D). In contrast, SCAT is robust to the positive correlation structure and still displays a desirable behavior on the control of type I error (Figures 2C,D). Because of the failure in the type I error control, in the following we no longer consider the Fisher’s method.

The estimated statistical power is shown in Figures 2E,F. Here, several pronounced observations need to emphasize. **First**, SCAT substantially outperforms any individual one-tissue FUSION in our simulation settings (Figure 2E vs. Figure 2F). **Second**, as anticipated, ignoring correlation among *p*-values can indeed lead to power reduction. For example, the oracle TWAS (denoted by oracle in Figure 2F), which considers the true correlation among the test statistics, has an approximately 10.1% higher power compared with SCAT (denoted by SCAT13 in Figure 2F), and the advantage of the oracle TWAS would be more evident if less FUSION analyses are combined by SCAT (e.g., oracle vs. SCAT4 or oracle vs. SCAT8 in Figure 2F). However, as aforementioned, the oracle TWAS cannot be applicable due to unavailability of correlation structure in practice, while SCAT is a universal combination approach without such limitation.

**Third**, SCAT that combines FUSION with a larger set of tissues is often much more powerful than that contains a smaller set of tissues (e.g., SCAT13 vs. SCAT8 or SCAT4; here the number attached represents the number of tissues used in the SCAT analysis, with a greater number indicating more tissues included); in the extreme case where only one tissue in each group (i.e., SCAT1), SCAT reduces to FUSION and exhibits the similar behavior to FUSION. Note that, this simulation is also equivalent to the case where missing *p*-values emerge. Nevertheless, SCAT is still better than any FUSION analysis with one tissue as long as more than two significant tissues are contained. **Fourth**, however, it is not necessarily the case that SCAT can always improve the power. For example, we find SCAT would encounter a loss of power if some of the combined individual FUSION analyses are non-significant (Supplementary Figure S3). **Fifth**, it is shown that SCAT would loss the power as the increase in the correlation under various correlation structures (Supplementary Figure S4). For instance, SCAT has a power of 0.241, 0.317, 0.427, or 0.572 when the correlation is 0.9, 0.6, 0.3 or 0 in the exchangeable structure (Supplementary Figure S4A). In addition, as can be expected, different correlation structures among the test statistics have various influences on the power of SCAT (Supplementary Figures S4A–C).

### Associated Genes With ALS Discovered in Previous GWASs

**FIGURE 3:**
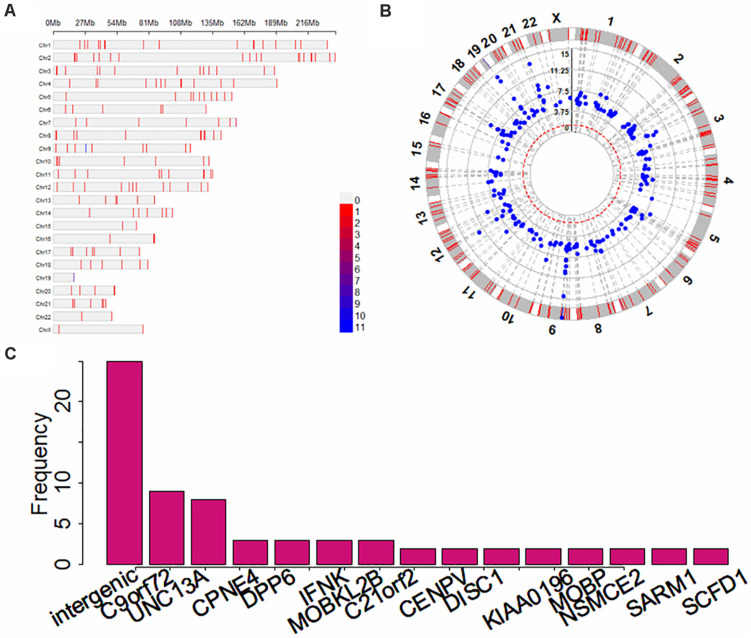
Summary results for ALS-associated SNPs and mapped genes identified in previous GWASs. **(A)** The distribution for associated SNPs across all 22 chromosomes; **(B)** The *p*-values of circle Manhattan plot of associated SNPs for significance; **(C)** The distribution for genes with high frequency.

In terms of the GWAS Catalog^1^, most of the ALS GWASs (17 out of 22) were performed on European individuals (Table 1). Totally, there are 313 SNP association pairs discovered across all chromosomes, especially in chromosomes 1 (i.e., 19 SNPs), 2 (i.e., 19 SNPs), and 9 (i.e., 21 SNPs) (Figures 3A,B). Those genetic variants are mapped to 253 unique genetic regions, among which 25 are located within *intergenic* (Figure 3C). In particular, *C9orf72* — a famous risk gene of ALS (Renton, 2011; Byrne, 2012; Garcia-Redondo, 2013; Diekstra et al., 2014; Chen Y. et al., 2016) — is the most frequent gene. The remaining genes with high frequency include *UNC13A* and *CPNE4* (Figure 3C).

### Associated Genes With ALS Discovered by FUSION and SCAT

Now we applied FUSION to ALS using 13 GTEx brain tissues as reference transcriptome datasets and then combined the results with SCAT for the overall significance. The correlation among gene expressions is displayed in Supplementary Figure S5. A total of 11,469 unique genes are analyzed but only 361 overlapped genes emerging in all the 13 GTEx brain tissues. It is empirically demonstrated that the *p*-values of FUSION among various GTEx brain tissues exhibit highly positive dependency (Supplementary Figure S5), which, together the unavailability of correlation information makes nearly all previous *p*-values combined methods cannot be directly utilized.

**FIGURE 4:**
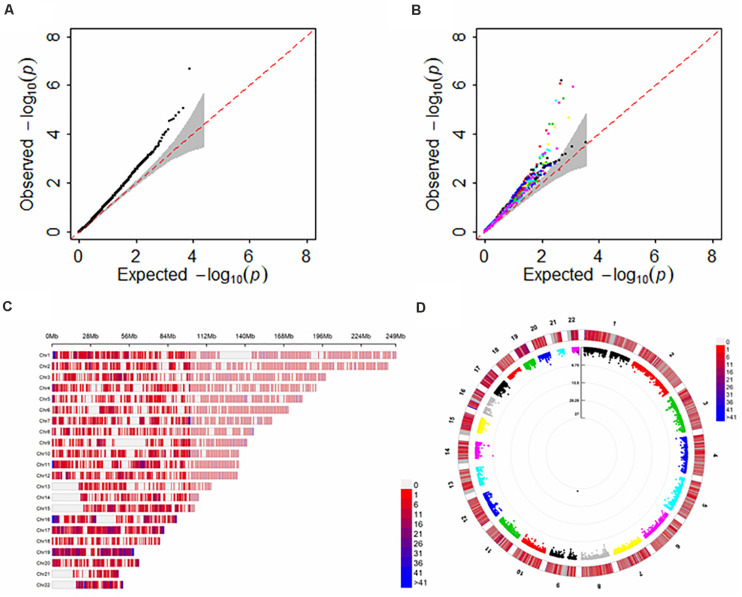
Results of FUSION and SCAT for TWAS analysis of ALS with multiple brain tissues. **(A)** The QQ plot for SCAT; **(B)** The QQ plot for FUSION with each of the GTEx brain tissues as reference dataset; **(C)** The distribution for analyzed genes across all 22 chromosomes; **(D)** The *p*-values of circle Manhattan plot of analyzed genes for significance. Of note, the genomic inflation factor of the p values obtained via SCAT is 1.04, indicating the slight inflation observed in **(A)** might be due to the polygenicity of ALS rather than uncontrolled unknown confounders.

For each GTEx brain tissue the number of genes with FDR < 0.05 (before adjustment of the issue of multiple tissues) is shown in Table 2 and Supplementary Figure S6A. The full results of TWAS for ALS are shown in Figure 4. It is seen that more genes are discovered in cerebellar hemisphere (i.e., 6 genes), following by anterior cingulate cortex BA24 and cerebellum (e.g., 4 genes for both tissues). Again, we observe that *C9orf72* is discovered to be associated with ALS in almost brain tissues which previously had been kept after screening of heritable genes in FUSION. However, if further considering the issue of multiple testing, many of these genes identified by single-tissue FUSION would be non-significant, leaving only two statistically significant genes (i.e., *SCFD1* and *C9orf72*).

The adjusted associations are displayed in Table 2 and Supplementary Figure S6B. Here, a total of eight genes are found by SCAT (FDR < 0.05), among which three (i.e., *SCFD1* with FDR = 0.001, *ATXN3* with FDR = 0.04 and *C9orf72* with FDR = 1.08E-22) are previously identified (Supplementary Table S1), while five (i.e., *SLC9A8* with FDR = 0.04, *FAM66D* with FDR = 0.04, *TRIP11* with FDR = 0.03, *JUP* with FDR = 0.04 and *RP11-529H20.6* with FDR = 0.04) are not. Except for *FAM66D* (antisense) and *RP11-529H20.6* (sense overlapping), all others are protein-coding genes (Supplementary Table S2). Furthermore, we find that there are no significant SNPs (with *p* < 5.00E-8) included within any of these five genes (Supplementary Figure S7). Thus, in our analysis *SLC9A8*, *FAM66D*, *TRIP11*, *JUP*, and *RP11-529H20.6* can be deemed to be newly genes that are likely associated with ALS.

## Discussion

Given the severe health threat and little knowledge of ALS, persistent work should be done to explore genetic and environmental risk factors related to ALS. The present study is one of such efforts with the aim to discover newly causal genes for ALS. To achieve this goal, we conducted the TWAS analysis and integrated association signals from multiple GTEx brain tissues to improve power by borrowing the idea of *p*-values combination. As demonstrated before, the main challenge in our TWAS analysis of ALS emerges in two aspects. First, multiple brain tissues were involved and the statistics of FUSION across tissues exhibited highly positive correlation; second, the dependency structure was unknown in practice because only summary-level statistics results can be available. Those difficulties lead to the failure of the Fisher’s method and also hamper the use of other commonly employed methods that can combine dependent *p*-values such as the Brown’s method (Brown, 1975), the Kost’s method (Kost and McDermott, 2002) and some tests proposed recently (Barnett et al., 2017; Gaynor et al., 2019; Sun et al., 2019; Sun and Lin, 2019), which typically require known covariance among *p*-values.

Our TWAS analysis relies on the newly flexible statistical framework of SCAT for hypothesis testing. Compared with FUSION (i.e., the summary-level TWAS analysis with one tissue each time), SCAT is more efficient as it aggregates individual association signals. With simulation studies we revealed that SCAT produced well-calibrated *p*-value for type I error control and was often much more powerful to identify associated signals across various scenarios compared with FUSION with only single tissue. Using SCAT we replicated three GWAS-discovered genes including *SCFD1* found in van Rheenen et al. (2016) and Nicolas et al. (2018), *ATXN3* identified in Nicolas et al. (2018) and *C9orf72* found in multiple previous GWASs (Supplementary Table S1). Among those *C9orf72* is a well-known genetic mutation of ALS previously detected in both European population (The Alsgen Consortium, 2013; Diekstra et al., 2014; McLaughlin et al., 2015; van Rheenen et al., 2016; Nicolas et al., 2018; Dekker et al., 2019) and East Asian population (Benyamin et al., 2017).

More importantly, with SCAT we identified five newly ALS-associated genes that were otherwise missed by existing statistical strategies, including *SLC9A8*, *FAM66D*, *TRIP11*, *JUP*, and *RP11-529H20.6*. Our new findings are also partially supported by previous studies. First, in the molecular level one typical pathological hallmark for neurodegeneration of ALS (e.g., tau, amyloid, and beta-protein precursor) is the change in cell cycle control and progression, which can be regulated by *SLC9A8* by inhibiting Na^+^/H^+^ exchanger activity in epithelia (Hu et al., 1998; Orlowski and Grinstein, 2004). In the population level, *SLC9A8* exhibits widely pleiotropic influence on chronic inflammatory diseases including ankylosing spondylitis, Crohn’s disease, psoriasis, primary sclerosing cholangitis, and ulcerative colitis (Stuart et al., 2010; Ellinghaus et al., 2016); in addition, *SLC9A8* is also associated with psoriasis (Stuart et al., 2010), gut microbiota (beta diversity) (Wang et al., 2016) and multiple sclerosis (International Multiple Sclerosis Genetics Consortium, 2013).

Second, *TRIP11* can provide instruction for generating a type of protein known as Golgi microtubule-associated protein 210 (GMAP-210) (Infante et al., 1999). This protein is found in the Golgi apparatus, a cell structure in which newly produced proteins are modified so they can be activated. On the other hand, the depletion of Golgi matrix proteins can result in an abnormal, fragmented Golgi morphology, which has been observed in multiple neurodegenerative diseases including ALS (Fujita and Okamoto, 2005), suggesting that the fragmentation of Golgi apparatus may be related to the neuronal degeneration of ALS. In population-based studies, *TRIP11* is identified to be associated with anthropometric traits including height (Gudbjartsson et al., 2008; Lettre et al., 2008; Lango Allen et al., 2010; Wood et al., 2014; He et al., 2015; Tachmazidou et al., 2017; Akiyama et al., 2019) and waist circumference adjusted for body mass index (Shungin et al., 2015; Graff et al., 2017; Justice et al., 2017), which are in turn believed to be relevant to the development of ALS (Desport et al., 1999; Jawaid et al., 2010; Paganoni et al., 2011; Shimizu et al., 2012; O’Reilly et al., 2013; Reich-Slotky et al., 2013; Calvo et al., 2017; Peter et al., 2017; Zeng et al., 2019b).

Third, *JUP* can regulate plakoglobin, a protein plays an important role in signaling within cells as part of the Wingless/Int (Wnt) pathway (Asimaki et al., 2007). The Wnt is a key pathway involved in neural development during embryogenesis (Wang and Wynshaw-Boris, 2004; Harrison-Uy and Pleasure, 2012) and in the maintenance of neuronal homeostasis (Ille and Sommer, 2005; Zhang et al., 2011). In particular, the perturbations of the Wnt pathway have been shown to have a correlation to neurological disorders (De Ferrari and Moon, 2006) as well as neurodegenerative diseases (De Ferrari et al., 2003; Inestrosa and Arenas, 2010).

In addition, in terms of BioSystems *SLC9A8* and *TRIP11* belong to the pathway of GO 0000139 Golgi membrane and *JUP* belongs to the pathway of GO 0000988 transcription factor activity, both of which have a functional role on brain tissues. All those provide evidence that supports the relationship between *SLC9A8*, *JUP*, and *TRIP11* with ALS. It also suggests that those genes may be associated with ALS in a direct, pleiotropic or mediated manner. Those new discoveries are expected to have the potential to advance our understanding of the molecular mechanism with regards to ALS and offer new insight into the etiology of ALS.

Besides discovering new ALS-associated genes, another contribution of the present study exists in the development of SCAT that can integrate a series of correlated association signals efficiently. As illustrated before, SCAT owns the attractive advantage that it takes the summary of a group of *p*-values as test statistic and evaluates the significance analytically without the knowledge of correlation structure (Liu et al., 2019; Liu and Xie, 2019). Therefore, as enthusiastic interest in TWAS continues to grow with more and more genetic and transcriptome data sets collected, especially since large scale individual-level datasets are still unable to obtain for some reasons, we believe that SCAT possesses extensive usefulness to many analogous situations of integrative genomic analyses.

Finally, several limitations of our work need to state. First, among the five new SCAT-identified genes, we do not find reasonable evidence for *FAM66D* and *RP11-529H20.6* in the literature. Second, we cannot replicate those new discoveries in external data sets since such data resources are unavailable for us; we thus simultaneously highlight the need to further validate our findings with additional investigation and experimental follow-up. Third, the used GTEx brain transcriptome reference panels have small samples sizes (ranging from 70 to 137, with the average of 102); as a result, our TWAS analysis may have only limited power. Nevertheless, we note that, in terms of the number of associated genes detected by FUSION with single brain tissue, we believe those new associations are more likely biologically relevant to ALS rather than completely driven by tissues with greater sample size. For example, only 0.07% (i.e., 4) genes were found in brain cerebellum although it has the largest sample size (i.e., 137) and the greatest *cis*-heritable genes (i.e., 5,752); while 0.15% genes were identified in brain anterior cingulate cortex BA24 which has only moderate sample size (i.e., 102) and *cis*-heritable genes (i.e., 2,653). Fourth, because not all genes can be available across all GTEx brain tissues (e.g., Table 2), we cannot determine ALS-specific tissues or identify tissue-specific ALS-associated genes, although both are also very interesting and worth of pursuing further (Sonawane et al., 2017; Finucane et al., 2018; Hao et al., 2018). Nevertheless, results displayed in Table 2 offer some suggestive observations for this issue. For instance, *FAM66D* is likely specially associated with ALS in brain cortex and *RP11-529H20.6* is possibly specifically associated with ALS in brain nucleus accumbens basal ganglia; *ATXN3*, *SCFD1* and *SLC9A8* are relevant to ALS in some brain tissues but not others; while *C9orf72* is associated with ALS across nearly all brain tissues. We note that the step-down inference procedure introduced in Sun et al. (2019) may be a promising approach that can be applied to discriminate which genes drive the observed association signal; but we reserve this problem for investigation in the future.

## Data Availability Statement

The raw data supporting the conclusions of this article will be made available by the authors, without undue reservation.

## Author Contributions

PZ conceived the idea for the study. PZ, LX, SH, and ZY obtained the data. TW and PZ cleared up the datasets. PZ, LX, SJ, and ZY performed the data analyses. PZ, LX, and ZY interpreted the results of the data analyses. PZ, LX, and ZY drafted the manuscript. All the authors approved the manuscript and provided relevant suggestions.

## Conflict of Interest

The authors declare that the research was conducted in the absence of any commercial or financial relationships that could be construed as a potential conflict of interest.

## Footnotes

1 https://www.ebi.ac.uk/gwas
