# Supplementary material for: Multiple-Tissue Integrative Transcriptome-Wide Association Studies Discovered New Genes Associated With Amyotrophic Lateral Sclerosis
Source: Front Genet. 2020 Nov 20;11:587243. doi: 10.3389/fgene.2020.587243 (PMC7714931; doi:10.3389/fgene.2020.587243)
Supplement: Supplementary file 1 [file Data_Sheet_1.docx]

**Supplementary File**


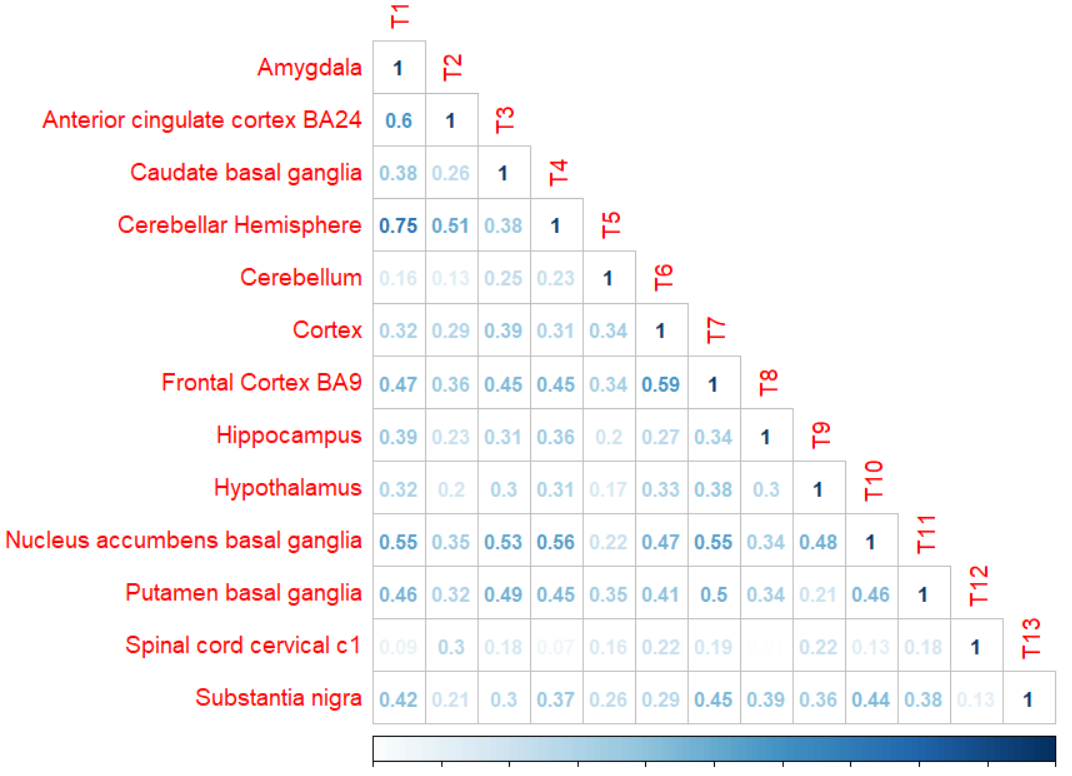


**Figure S1** Correlation matrix of gene expressions estimated from 13 GTEx brain tissues across all available genes. We here included 11 donators who had complete gene expressions across those tissues. After removing all zero-expressed genes in any donator, we performed the TMM normalization method [[1](#_ENREF_1)] on read counts for the remaining 15,157 genes using the cpm function (with prior count equal to zero and log2 transformation) in the edgeR package [[**2**](#_ENREF_2)]. The correlation matrix was computed in a shrinkage fashion [[3-5](#_ENREF_3)]: $R \text{=} \text{λ} \times\hat{R} \text{+} (1 - \text{λ)} \times I$ with $\hat{R}$ the empirical correlation matrix among gene expressions and λ = 0.9.


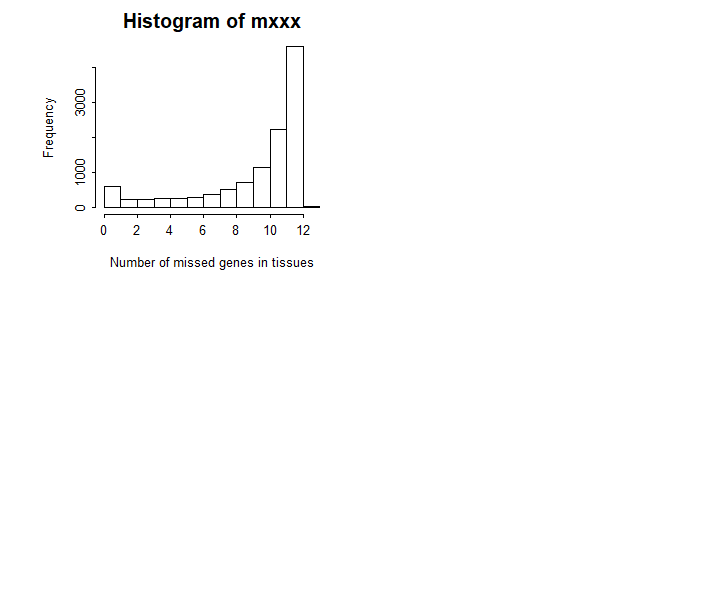


**Figure S2** Number of missing genes in the TWAS analysis with FUSION across 13 GTEx brain tissues. For example, there are 236 genes having one missing *p*-value, 233 genes having two missing *p*-values; and so forth.


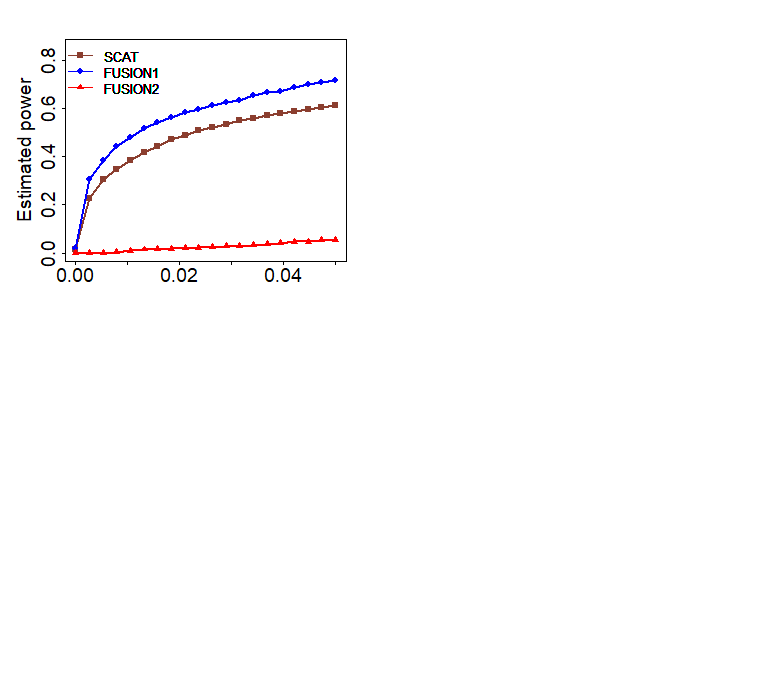


**Figure S3** Estimated statistical power of SCAT when combining two FUSION analyses and one of them is non-significant. Specifically, a two-dimensional vector was generated from a multivariate normal distribution with the mean being (2.5, 0) and the variance matrix being $\left(\begin{matrix} 1 & 0.5 \\ 0.5 & 1 \end{matrix}\right)$; and then we produced the p values in terms of the standard normal destitution. That is, here one of the p values obtained from individual FUSION analyses is always non-significant. Here, we clearly find that SCAT encounters a power reduction because redundant p values are combined.


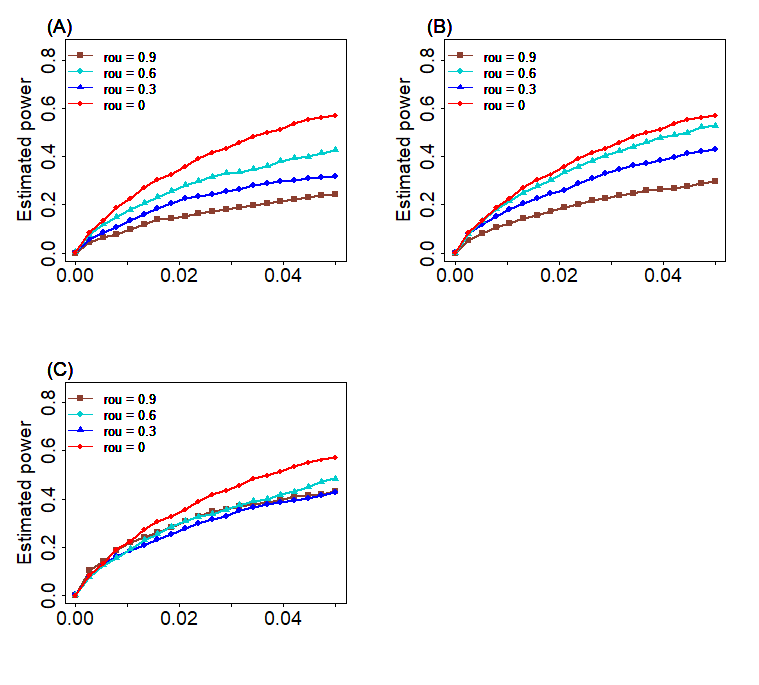


**Figure S4** Estimated statistical power of SCAT under various correlation structures. Here, as done in the simulations in the main text, we obtained p values based on Z values. Specifically, we first obtained the correlation matrix of **Z** values of FUSION (i.e. the **C** matrix) and generated a 13-dimentional multivariate random variable which followed **MVN**(***μ***, **C**); then, we yielded the *p*-value for each marginal random variable by assuming it followed a standard normal distribution. Finally, we combined these *p*-values with SCAT. For a fair comparison, we set each element of ***μ*** to be $\sqrt{3\log(T)}/T^{(1/3)}$ with *T* = 13. Three correlation structures were considered, including the exchangeable structure (**A**), the autoregressive structure (**B**) and the m-dependent structure (**C**). We varied the correlation in these structures to assess the power performance of SCAT.


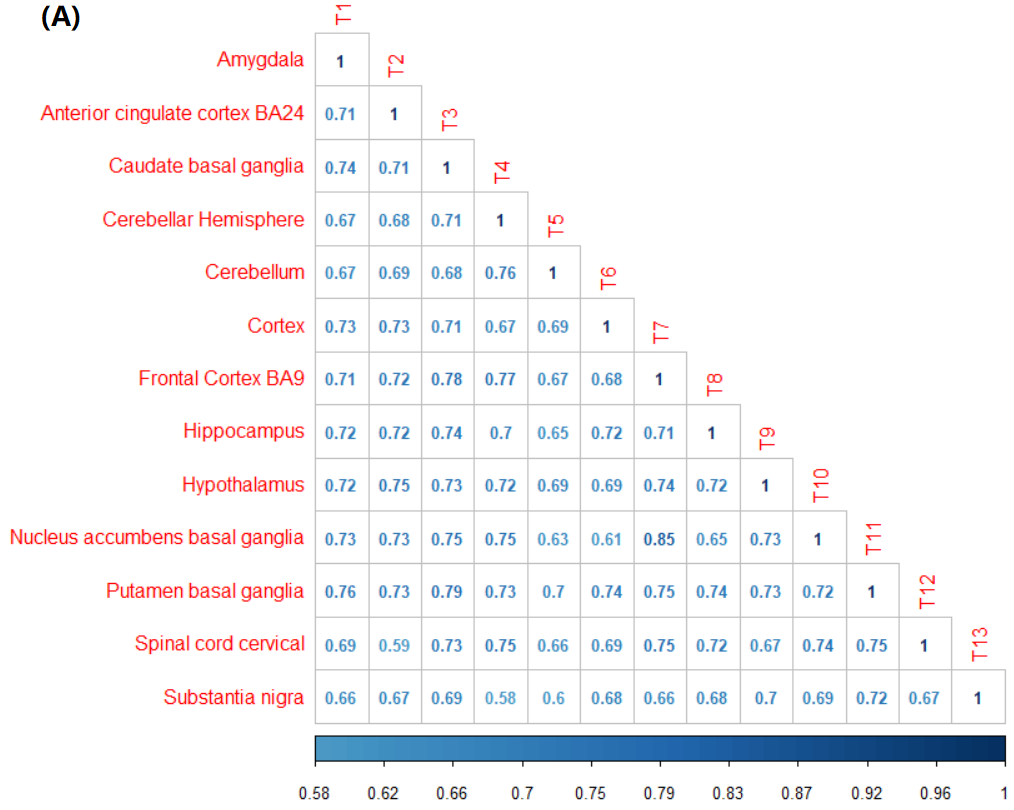


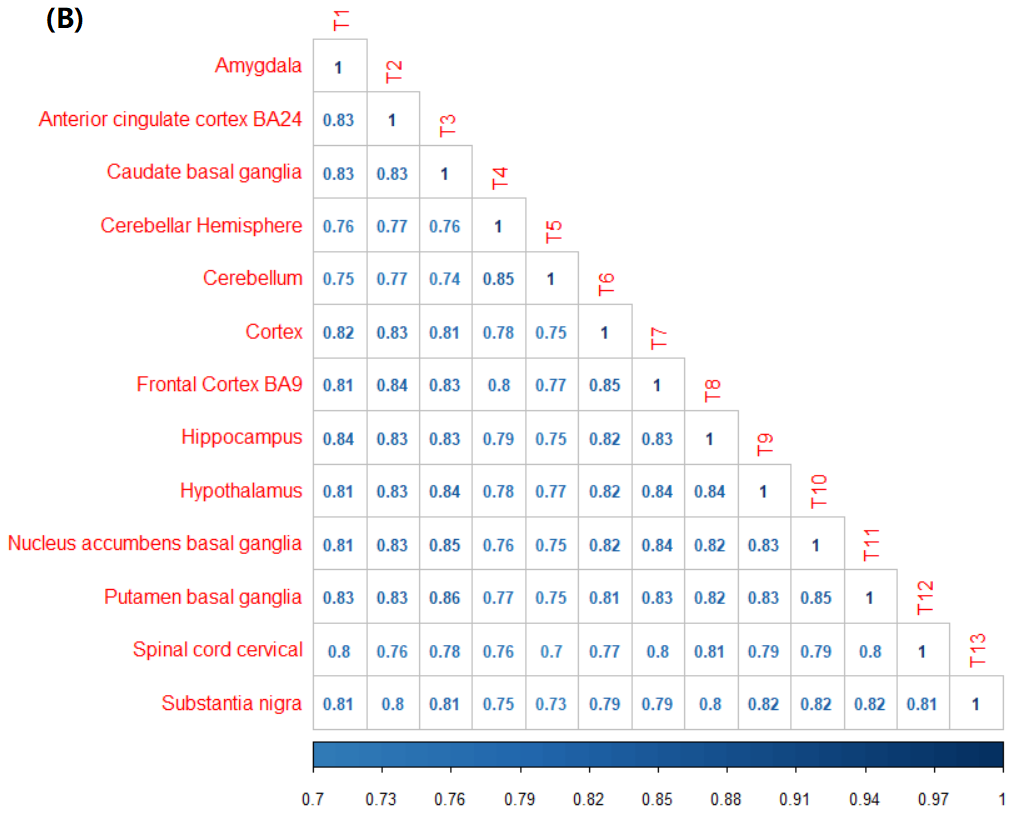


**Figure S5 (A)** Correlation matrix of *p*-values in -log(10) scale for FUSION from 13 GTEx brain tissues across genes. **(B)** Correlation matrix of Z scores for FUSION from 13 GTEx brain tissues across genes.

(A) (B)


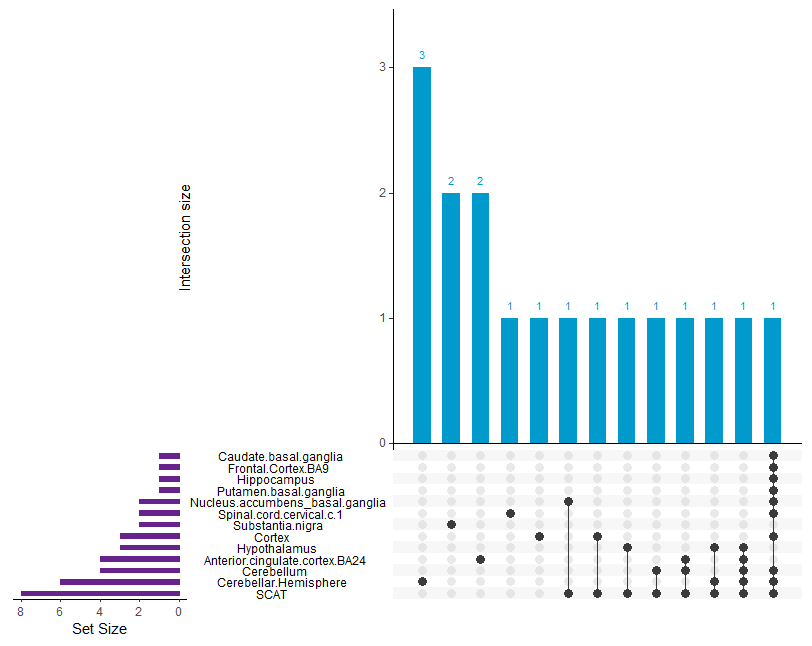

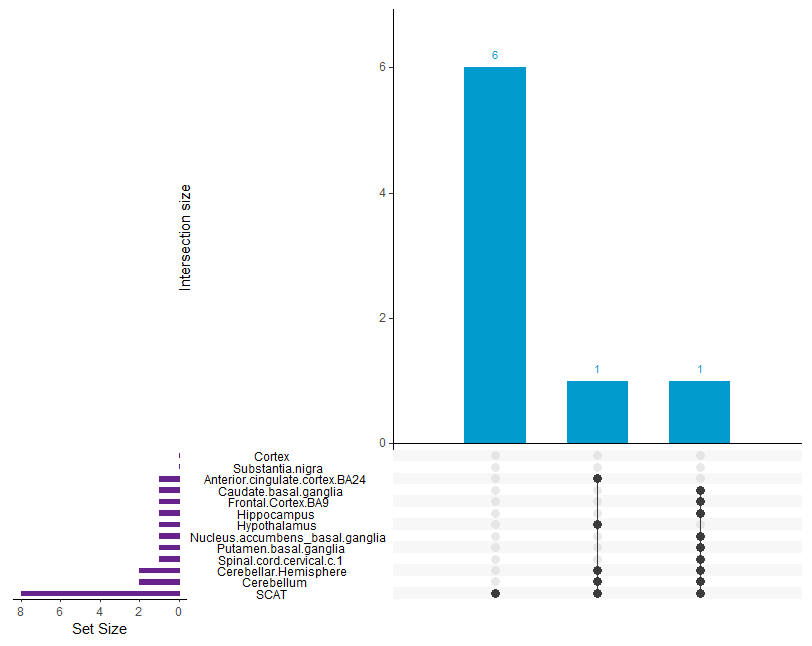


**Figure S6** Upset plot of genes identified by FUSION (i.e. TWAS with single tissue) and SCAT for ALS from 13 GTEx brain tissues across genes. (**A**) the shared genes before the adjustment of multiple comparison; (**B**) the shared genes after the adjustment of multiple comparison. Each bar in the plot represents the number of shared genes discovered in various tissues or detected by SCAT. The Upset plot was created by the R UpSetR package [[6](#_ENREF_6)].

**Figure S7** LocusZoom plots for eight genes associated with ALS identified by SCAT. Those plots were generated with the online tool at <http://locuszoom.org/> [[7](#_ENREF_7)].

**Table S1** Association studies for ALS in terms of the GWAS catalog

| year | reported genes | Reference |
| --- | --- | --- |
| 2007 | *KIAA1727, SUSD1, ZFP64* | [[8](#_ENREF_8)] |
| 2007 | *ITPR2* | [[9](#_ENREF_9)] |
| 2007 | *DPP6* | [[10](#_ENREF_10)] |
| 2008 | *DPP6, LIPC, ITPR2* | [[11](#_ENREF_11)] |
| 2009 | *DPP6* | [[12](#_ENREF_12)] |
| 2009 | *ATXN1,B4GALT6,CNTN4, CSNK1G3,DISC1,EFEMP1,KIFAP3,NT5C1A,RBMS1, SCN7A,SELL,*  *SEMA6A,SLC39A11,ZNF746* | [[13](#_ENREF_13)] |
| 2009 | *IFNK, MOBKL2B, C9orf72* | [[14](#_ENREF_14)] |
| 2010 | *IFNK, MOBKL2B, C9orf72, SOD1* | [[15](#_ENREF_15)] |
| 2010 | *intergenic* | [[16](#_ENREF_16)] |
| 2010 | *LOC100506746, OR52K1* | [[17](#_ENREF_17)] |
| 2013 | *C9orf72,CPNE4,KIAA0513,LAMA3,PIGL, CENPV,STK36, TTLL4, ZNF142*  *UNC13A,ANKS1B,ARAP2,ARHGEF2,CNOT2,IFRD1,JMJD2A,NRXN3,*  *PCSK5,PTPRF,ST3GAL3* | [[18](#_ENREF_18)] |
| 2013 | *CAMK1G,SUSD2, CABIN1, GGT5* | [[19](#_ENREF_19)] |
| 2013 | DYRK2, CAND1,EGR1, REEP2,SEC16B,BOD1L,CREB5,  LRRC3, C21orf29,TBC1D1,CPNE4,HDAC4, ASB1,PPP2R2D,UNC13A | [[18](#_ENREF_18)] |
| 2013 | *ANKRD29,SALM1,UNC13A* | [[20](#_ENREF_20)] |
| 2013 | *ABCC12,ABCG1,ACCN1,ADAMTS18,ADAMTSL1,ADAMTSL3,AGXT2L1,ALCAM,ALDH3A1,*  *ANK3,ANXA3, SB13,ATP2B2,AUTS2,BEND7,BMPR1B,BRUNOL4,C16orf74,C18orf58,C20orf173,*  *C21orf131,C22orf34,C3orf56,C5orf15,C6orf132,C9orf27,CALN1,CD2AP,CEP250,CHD2,*  *CHODL,CNTLN,CNTN5,CRBN,CSMD1,CSNK1A1L,CTNND2,CX3CR1,CXCR4,DACH1,*  *DISC1,DKFZp434E1119,DOCK4,DPF1,EPB41,ERBB4,ERG,FAM119A,FAM167A,FAM19A1,*  *FAM5C,FANK1,FAT3,FBXO15,FGF9,FLI1,FYN,GLRX5,GNA14,GPR133,HADH,HOXC13,*  *HOXD10,IFT74,INPP4B,INPP5B,INTS6,IQCF5,ITGA9,KC6,KCNMB2,KCTD16,KIAA0182,*  *KIAA0947,KIAA1680,LAMA2,LDHC,LOC100128095, PCML,LOC100129986,LOC100130298,*  *LOC100286951,LOC100287135,LOC100287172,LOC100287306,LOC100287580,LOC100288911*  *,LOC100289178,LOC100289459,LOC152118,LOC387820,LOC400750,LOC645314,LOC645321,*  *LOC727677,LOC728755,LOC729204,LPIN2,LRRC8C,LRRTM4,MACROD2,MAP3K7,MASP1,*  *MAT2B,MGLL,MRAS,MSC,MYO18B,NEDD4L,NEUROG2,NFASC,NFATC2,NKAIN3,NOG,*  *NR3C2,NTRK3,NUDT12,OLFM4,PAX3,PDGFRL,PFKP,PLIN,PPP2R5D,PROCR,PRR20,PTH2R,*  *PVT1,RAB3GAP2,RAB9P1,RBM19,RGS6,RNF165,RNF19A,RP1L1,RPS6KA1,RTCD1,RYR3,SDC1,*  *SEC16B,SLC10A2,SLC25A12,SLC28A3,SLC5A8,SLCO2A1,SLITRK1,SMARCA2,SNORD114-31,*  *SNX19,SP4,SREBF2,SSTR4,ST6GALNAC5,STON1,STOX1,SYNPO2,SYT16,TAPT1,TBC1D1,TBXAS1,*  *TFAP2A,THRB,TLL1,TMEM132B,TMEM132E,TMPRSS2,TP53I11,TRPM8,TSPAN9,TTC15,TYRP1,*  *WAPAL,WWC2,ZFYVE26,ZNF28,ZNF354A,ZNF700* | [[21](#_ENREF_21)] |
| 2014 | SQLE, NSMCE2, KIAA0196,  C9orf72,CENPV,SQLE, KIAA0196,  NSMCE2,TXNDC6,UNC13A | [[22](#_ENREF_22)] |
| 2015 | C9orf72 | [[23](#_ENREF_23)] |
| 2016 | KALRN | [[24](#_ENREF_24)] |
| 2016 | C21orf2,C9orf72,MOBP,SARM1,  SCFD1,TBK1,UNC13A | [[25](#_ENREF_25)] |
| 2017 | c9orf72,MOBP,SARM1,UNC13A | [[26](#_ENREF_26)] |
| 2018 | ATXN3,C21orf2,C9orf72,KIF5A,LOC101927815,  PMP22,SCFD1,TBK1,TNIP1,UNC13A | [[27](#_ENREF_27)] |
| 2019 | C9orf72 | [[28](#_ENREF_28)] |

The results were overviewed in terms of the GWAS catalog at <https://www.ebi.ac.uk/gwas> (until 2020-02-02).

**Table S2** Basic information for the eight genes associated with ALS identified by SCAT

| gene | chromosome | position | | gene type |
| --- | --- | --- | --- | --- |
|  |  | low | up |  |
| *FAM66D* | 8 | 11,973,284 | 12,008,698 | antisense |
| *C9orf72* | 9 | 27,546,544 | 27,573,864 | protein coding |
| *TRIP11* | 14 | 92,432,335 | 92,507,241 | protein coding |
| *RP11-529H20.6* | 14 | 92,511,119 | 92,516,990 | sense overlapping |
| *ATXN3* | 14 | 92,524,896 | 92,572,965 | protein coding |
| *SCFD1* | 14 | 31,091,318 | 31,205,018 | protein coding |
| *JUP* | 17 | 39,775,692 | 39,943,183 | protein coding |
| *SLC9A8* | 20 | 48,429,250 | 48,508,779 | protein coding |

# **References**

1. Robinson MD, Oshlack A: **A scaling normalization method for differential expression analysis of RNA-seq data**. *Genome Biol* 2010, **11**.

2. Robinson MD, McCarthy DJ, Smyth GK: **edgeR: a Bioconductor package for differential expression analysis of digital gene expression data**. *Bioinformatics* 2010, **26**(1):139-140.

3. Schäfer J, Strimmer K: **A shrinkage approach to large-scale covariance matrix estimation and implications for functional genomics**. *Stat Appl Genet Mol Biol* 2005, **4**.

4. Opgen-Rhein R, Strimmer K: **Accurate Ranking of Differentially Expressed Genes by a Distribution-Free Shrinkage Approach**. In: *Stat Appl Genet Mol Biol.* vol. 6; 2007.

5. Yang Y, Shi X, Jiao Y, Huang J, Chen M, Zhou X, Sun L, Lin X, Yang C, Liu J: **CoMM-S2: a collaborative mixed model using summary statistics in transcriptome-wide association studies**. *bioRxiv* 2019:652263.

6. Lex A, Gehlenborg N, Strobelt H, Vuillemot R, Pfister H: **UpSet: Visualization of Intersecting Sets**. *IEEE Transactions on Visualization and Computer Graphics* 2014, **20**(12):1983-1992.

7. Pruim RJ, Welch RP, Sanna S, Teslovich TM, Chines PS, Gliedt TP, Boehnke M, Abecasis GR, Willer CJ: **LocusZoom: regional visualization of genome-wide association scan results**. *Bioinformatics* 2010, **26**(18):2336-2337.

8. Schymick JC, Scholz SW, Fung H-C, Britton A, Arepalli S, Gibbs JR, Lombardo F, Matarin M, Kasperaviciute D, Hernandez DG: **Genome-wide genotyping in amyotrophic lateral sclerosis and neurologically normal controls: first stage analysis and public release of data**. *Lancet Neurol* 2007, **6**(4):322-328.

9. Van Es MA, Van Vught PW, Blauw HM, Franke L, Saris CG, Andersen PM, Van Den Bosch L, de Jong SW, van't Slot R, Birve A: **ITPR2 as a susceptibility gene in sporadic amyotrophic lateral sclerosis: a genome-wide association study**. *Lancet Neurol* 2007, **6**(10):869-877.

10. Cronin S, Berger S, Ding J, Schymick JC, Washecka N, Hernandez DG, Greenway MJ, Bradley DG, Traynor BJ, Hardiman O: **A genome-wide association study of sporadic ALS in a homogenous Irish population**. *Hum Mol Genet* 2007, **17**(5):768-774.

11. Van Es MA, Van Vught PW, Blauw HM, Franke L, Saris CG, Van Den Bosch L, De Jong SW, De Jong V, Baas F, Van't Slot R: **Genetic variation in DPP6 is associated with susceptibility to amyotrophic lateral sclerosis**. *Nat Genet* 2008, **40**(1):29-31.

12. Cronin S, Tomik B, Bradley DG, Slowik A, Hardiman O: **Screening for replication of genome-wide SNP associations in sporadic ALS**. *Eur J Hum Genet* 2009, **17**(2):213-218.

13. Landers JE, Melki J, Meininger V, Glass JD, van den Berg LH, van Es MA, Sapp PC, van Vught PW, McKenna-Yasek DM, Blauw HM: **Reduced expression of the Kinesin-Associated Protein 3 (KIFAP3) gene increases survival in sporadic amyotrophic lateral sclerosis**. *Proceedings of the National Academy of Sciences* 2009, **106**(22):9004-9009.

14. van Es MA, Veldink JH, Saris CGJ, Blauw HM, van Vught PWJ, Birve A, Lemmens R, Schelhaas HJ, Groen EJN, Huisman MHB *et al*: **Genome-wide association study identifies 19p13.3 (UNC13A) and 9p21.2 as susceptibility loci for sporadic amyotrophic lateral sclerosis**. *Nat Genet* 2009, **41**(10):1083-1087.

15. Laaksovirta H, Peuralinna T, Schymick JC, Scholz SW, Lai S-L, Myllykangas L, Sulkava R, Jansson L, Hernandez DG, Gibbs JR: **Chromosome 9p21 in amyotrophic lateral sclerosis in Finland: a genome-wide association study**. *Lancet Neurol* 2010, **9**(10):978-985.

16. Shatunov A, Mok K, Newhouse S, Weale ME, Smith B, Vance C, Johnson L, Veldink JH, van Es MA, van den Berg LH: **Chromosome 9p21 in sporadic amyotrophic lateral sclerosis in the UK and seven other countries: a genome-wide association study**. *Lancet Neurol* 2010, **9**(10):986-994.

17. Kwee LC, Liu Y, Haynes C, Gibson JR, Stone A, Schichman SA, Kamel F, Nelson LM, Topol B, Van Den Eeden SK: **A high-density genome-wide association screen of sporadic ALS in US veterans**. *PLoS ONE* 2012, **7**(3):e32768.

18. The ALSGEN Consortium: **Age of onset of amyotrophic lateral sclerosis is modulated by a locus on 1p34. 1**. *Neurobiol Aging* 2013, **34**(1):357. e357-357. e319.

19. Deng M, Wei L, Zuo X, Tian Y, Xie F, Hu P, Zhu C, Yu F, Meng Y, Wang H *et al*: **Genome-wide association analyses in Han Chinese identify two new susceptibility loci for amyotrophic lateral sclerosis**. *Nat Genet* 2013, **45**(6):697-700.

20. Fogh I, Ratti A, Gellera C, Lin K, Tiloca C, Moskvina V, Corrado L, Sorarù G, Cereda C, Corti S: **A genome-wide association meta-analysis identifies a novel locus at 17q11. 2 associated with sporadic amyotrophic lateral sclerosis**. *Hum Mol Genet* 2013, **23**(8):2220-2231.

21. Xie T, Deng L, Mei P, Zhou Y, Wang B, Zhang J, Lin J, Wei Y, Zhang X, Xu R: **A genome-wide association study combining pathway analysis for typical sporadic amyotrophic lateral sclerosis in Chinese Han populations**. *Neurobiol Aging* 2014, **35**(7):1778. e1779-1778. e1723.

22. Diekstra FP, Deerlin VM, Swieten JC, Al‐Chalabi A, Ludolph AC, Weishaupt JH, Hardiman O, Landers JE, Brown RH, Es MA: **C9orf72 and UNC13A are shared risk loci for amyotrophic lateral sclerosis and frontotemporal dementia: A genome-wide meta-analysis**. *Ann Neurol* 2014, **76**(1):120-133.

23. McLaughlin RL, Kenna KP, Vajda A, Bede P, Elamin M, Cronin S, Donaghy CG, Bradley DG, Hardiman O: **A second-generation Irish genome-wide association study for amyotrophic lateral sclerosis**. *Neurobiol Aging* 2015, **36**(2):1221.e1227-1221.e1213.

24. Chen C-J, Chen C-M, Pai T-W, Chang H-T, Hwang C-S: **A genome-wide association study on amyotrophic lateral sclerosis in the Taiwanese Han population**. *Biomark Med* 2016, **10**(6):597-611.

25. van Rheenen W, Shatunov A, Dekker AM, McLaughlin RL, Diekstra FP, Pulit SL, van der Spek RA, Vosa U, de Jong S, Robinson MR *et al*: **Genome-wide association analyses identify new risk variants and the genetic architecture of amyotrophic lateral sclerosis**. *Nat Genet* 2016, **48**(9):1043-1048.

26. Benyamin B, He J, Zhao Q, Gratten J, Garton F, Leo PJ, Liu Z, Mangelsdorf M, Al-Chalabi A, Anderson L: **Cross-ethnic meta-analysis identifies association of the GPX3-TNIP1 locus with amyotrophic lateral sclerosis**. *Nat Commun* 2017, **8**(1):611.

27. Nicolas A, Kenna KP, Renton AE, Ticozzi N, Faghri F, Chia R, Dominov JA, Kenna BJ, Nalls MA, Keagle P *et al*: **Genome-wide Analyses Identify KIF5A as a Novel ALS Gene**. *Neuron* 2018, **97**(6).

28. Dekker AM, Diekstra FP, Pulit SL, Tazelaar GHP, van der Spek RA, van Rheenen W, van Eijk KR, Calvo A, Brunetti M, Damme PV *et al*: **Exome array analysis of rare and low frequency variants in amyotrophic lateral sclerosis**. *Scientific Reports* 2019, **9**(1):5931.
